# The Fab fragment of anti-IgE Cε2 domain prevents allergic reactions through interacting with IgE-FcεRIα complex on rat mast cells

**DOI:** 10.1038/s41598-018-32200-z

**Published:** 2018-09-24

**Authors:** Takao Hirano, Akemi Koyanagi, Kaoru Kotoshiba, Yoichi Shinkai, Masataka Kasai, Tomoaki Ando, Ayako Kaitani, Ko Okumura, Jiro Kitaura

**Affiliations:** 1 Division of Hematology, Department of Internal Medicine, Department of General Medicine, Juntendo University Nerima Hospital, 3-1-10 Nerima, Takanodai, Nerima-ku, Tokyo Japan; 2 Laboratory of Cell Biology, Research Support Center, Juntendo University Graduate School of Medicine, 2-1-1 Hongo, Bunkyo-ku, Tokyo Japan; 3 Cellular Memory Laboratory, RIKEN, 2-1 Hirosawa, Wako, Saitama 351-0198 Japan; 4 Atopy (Allergy) Research Center, Juntendo University Graduate School of Medicine, Juntendo University Graduate School of Medicine, 2-1-1 Hongo, Bunkyo-ku, Tokyo Japan

## Abstract

Immunoglobulin E (IgE) plays a central role in the pathogenesis of Type I hypersensitivity through interaction with a high-affinity receptor (FcεRIα). For therapeutic applications, substantial attention has been focused recently on the blockade of the IgE interaction with FcεRIα. While exploring better options for preventing allergic diseases, we found that the Fab fragment of the rat anti-murine IgE antibody (Fab-6HD5) strongly inhibited passive cutaneous anaphylaxis (PCA) *in vivo*, as well as spleen tyrosine kinase (Syk) activity and β-hexosaminidase release from basophilic leukemia cells *in vitro*. The *in vivo* effects of Fab-6HD5 pre-administration were maintained over a long period of time for at least 10 days. Using flow cytometry analysis, we also found that Fab-6HD5 did not recognize the IgE Cε3 domain containing specific binding sites for FcεRIα. Furthermore, deletion-mapping studies revealed that Fab-6HD5 recognized conformational epitopes on the Cε2 domain of IgE. Given that the Cε2 domain plays a key role in stabilizing the interaction of IgE with FcRIα, our results suggest that the specific binding of Fab-6HD5 to the Cε2 domain prevents allergic reactions through destabilizing the preformed IgE-FcεRIα complex on rat mast cells. Although the present study was performed using animal models, these findings support the idea that a certain antibody directed against IgE CH domains may contribute to preventing allergic diseases through interacting with IgE-FcεRIα complex.

## Introduction

Allergic diseases, including asthma, are the most common chronic diseases, and their prevalence has increased worldwide in recent decades^1^. In general, quality of life is often impaired in patients with asthma and allergic rhinitis. Immunoglobulin E (IgE), which was originally discovered in 1966 by Ishizaka *et al*.^2,3^, plays a pivotal role in allergic diseases, such as hay fever, food allergies, atopic dermatitis, asthma and anaphylaxis^4–6^. IgE binds to a high-affinity receptor (FcεRIα) on the surface of mast cells and basophils^7^. Cross-linking of IgE bound to FcεRIα with multivalent allergens results in the release of histamine and other chemical mediators that act on the surrounding tissues and the most common symptoms of Type I hypersensitivity^8–10^. Given that histamine plays a key role in inflammation, the use of antagonists targeting the histamine H1 and H4 receptor has been shown to be effective for the treatment of allergic diseases^11,12^. Allergen-specific immunotherapy through restoring immune tolerance to allergens has also been a widely used therapeutic approach^13^. Among potential treatments, anti-IgE therapy, which involves the neutralization of IgE by an anti-IgE antibody, appears to be the most promising strategy to treat allergic diseases to date. Indeed, omalizumab is the most widely used anti-IgE antibody drug^14–18^, and it leads to rapid and dramatic falls in free serum IgE levels as well as FcεRIα expression in mast cells^19,20^. Its clinical effects have been demonstrated in allergic diseases, such as asthma and chronic urticaria^16,21–24^. Because omalizumab consists of an intact, whole IgG molecule, its use in clinical trials showed several adverse effects, including two cases of anaphylaxis^25,26^. Undigested anti-IgE receptors and its F(ab’)_2_ induce a significant histamine release from mast cells, whereas the binding of Fab’ monomer fragments with receptors fails to do so^27^, which suggests that the Fab fragment of IgE reduces the risk of anaphylaxis. To analyze the anaphylactic reactions elicited by IgE antibodies, we previously produced rat monoclonal antibodies that react with murine IgE. In the present study, we focused on one of these antibodies (6HD5) and analyzed its mechanism of action for preventing allergic reactions.

## Results and Discussion

### Inhibition of IgE-mediated anaphylactic reactions by Fab-6HD5

To explore all avenues for preventing allergic diseases using anti-IgE therapy, we focused on a Fab fragment of a monoclonal anti-IgE antibody (Fab-6HD5) and investigated its *in vivo* effects on IgE-mediated anaphylactic reactions using a passive cutaneous anaphylaxis (PCA) assay. First, we injected serial dilutions of anti-dinitrophenyl (DNP) IgE (SPE-7)^28^ intradermally into rats. Twenty-four hours later, several dilutions of anti-IgE antibodies (6HD5, Fab-6HD5, HMK-12, and Fab-HMK-12, with rat IgG as a negative control) were injected into the same sites. Following an extravasation assay with Evans blue and DNP-BSA, the results revealed that a minimal amount of Fab-6HD5 or 6HD5 (1.25 μg/ml) could inhibit the PCA reactions (Table 1). By contrast, 4 times the amount of anti-IgE antibodies, such as HMK-12 and Fab-HMK-12 (5 μg/ml), was needed to inhibit the PCA reactions. In addition, a significant inhibition of the PCA reaction by Fab-6HD5 was obtained for another allotype, anti-trinitrophenyl (TNP) IgE (142a). However, there was no inhibition of PCA reactions with Fab-anti-κ, which suggests that Fab-6HD5 is directed against an IgE H chain constant region. Previous studies have demonstrated that omalizumab inhibits the PCA reactions at concentration of 50 μM, but the inhibitory effect is less pronounced at 5 μM^29^. In contrast, it should be noted that a small amount of Fab-6HD5 (2 μg/ml) was sufficient to completely inhibit the PCA reactions.

**Table 1:** Inhibition of PCA by anti-IgE antibodies.

anti-DNPIgE vs	Antibodies, (μg/ml)						
	20	10	5	2.5	1.25	0.63	0
Fab-6HD5	−	−	−	−	±	+	+++
6HD5	−	−	−	−	+	++	+++
Fab-HMK12	−	−	+	++	++	+++	+++
HMK12	−	−	+	++	++	+++	+++
Rat IgG	+++	+++	+++	+++	+++	+++	+++
**anti-TNPIgE vs**	**Antibodies, (μg/ml)**						
	**20**	**10**	**5**	**2.5**	**1.25**	**0.63**	**0**
Fab-6HD5	−	−	−	−	±	+	+++
Fab-HMK12	−	−	+	++	++	+++	+++
Anti-κ	+++	+++	+++	+++	+++	+++	+++
Rat IgG	+++	+++	+++	+++	+++	+++	+++

Serial dilutions of anti-DNP IgE (SPE-7) or anti-TNP IgE (142a) were injected intradermally into rats. Twenty-four hours later, each antibody (Fab-6HD5, Fab-HMK-12, Fab-anti-κ, or rat IgG) was injected into the same site. Two hours after the second series of injections, the rats were injected intravenously with 0.5% Evans blue with DNP-BSA or TNP-BSA. The reactions (the diameters of the blue spots) were measured 30 min later. +++, blue spot of >15 mm in diameter; ++, ≥10 mm in diameter; +, <10 mm in diameter; ±, <5 mm in diameter; −, no visible blue spot.

### Long-term prevention of allergic reactions by Fab-6HD5

To gain further insights into the role of Fab-6HD5 in allergic reactions, we next investigated how long the inhibitory effects of Fab-6HD5 on PCA reactions lasted. In the first group, SPE-7 was injected intradermally into rats on day 0. Then, serial dilutions of Fab-6HD5 were injected into the same sites on day 1. Two hours later, Evans blue and DNP-BSA were injected intravenously for the challenge. In the second group, an Evans blue extravasation assay was performed on day 10. The results demonstrated that an inhibition of PCA reactions was observed at a concentration of Fab-6HD5 ranging from 0.63 μg/ml in the day-1 group and 0.32 μg/ml in the day-10 group (Table 2). These data clearly showed that inhibition of PCA reactions by this antibody lasts at least 10 days and maintains an even higher inhibition than the challenge on day 1. These findings raise an intriguing possibility that pretreatment with Fab-6HD5 may prevent allergic reactions over a long period of time. Furthermore, strong inhibition of PCA reactions was observed at 2 hours after the injection of Fab-6HD5, which suggests that this antibody has promising effects in the treatment of anaphylactic shock.

**Table 2:** Time course studies of PCA inhibition by 6HD5.

Evans Blue	Fab-6HD5, (μg/ml)								
extravasation	20	10	5	2.5	1.25	0.63	0.32	0.16	0
Day 1	−	−	−	−	±	±	+	++	+++
Day 10	−	−	−	−	−	±	±	+	+++

PCA day 1: SPE-7 (anti-DNP IgE) was injected intradermally into rats on day 0, and a serial dilution of Fab-6HD5 was injected into the same sites on day 1. Two hours later, an Evans blue extravasation assay was performed.

PCA day 10: an Evans blue extravasation assay was performed on day 10. +++, blue spot of >15 mm in diameter; ++, ≥10 mm in diameter; +, <10 mm in diameter; ±, <5 mm in diameter; −, no visible blue spot.

### Inhibitory effects of Fab-6HD5 on mast cell degranulation

**Figure 1:**
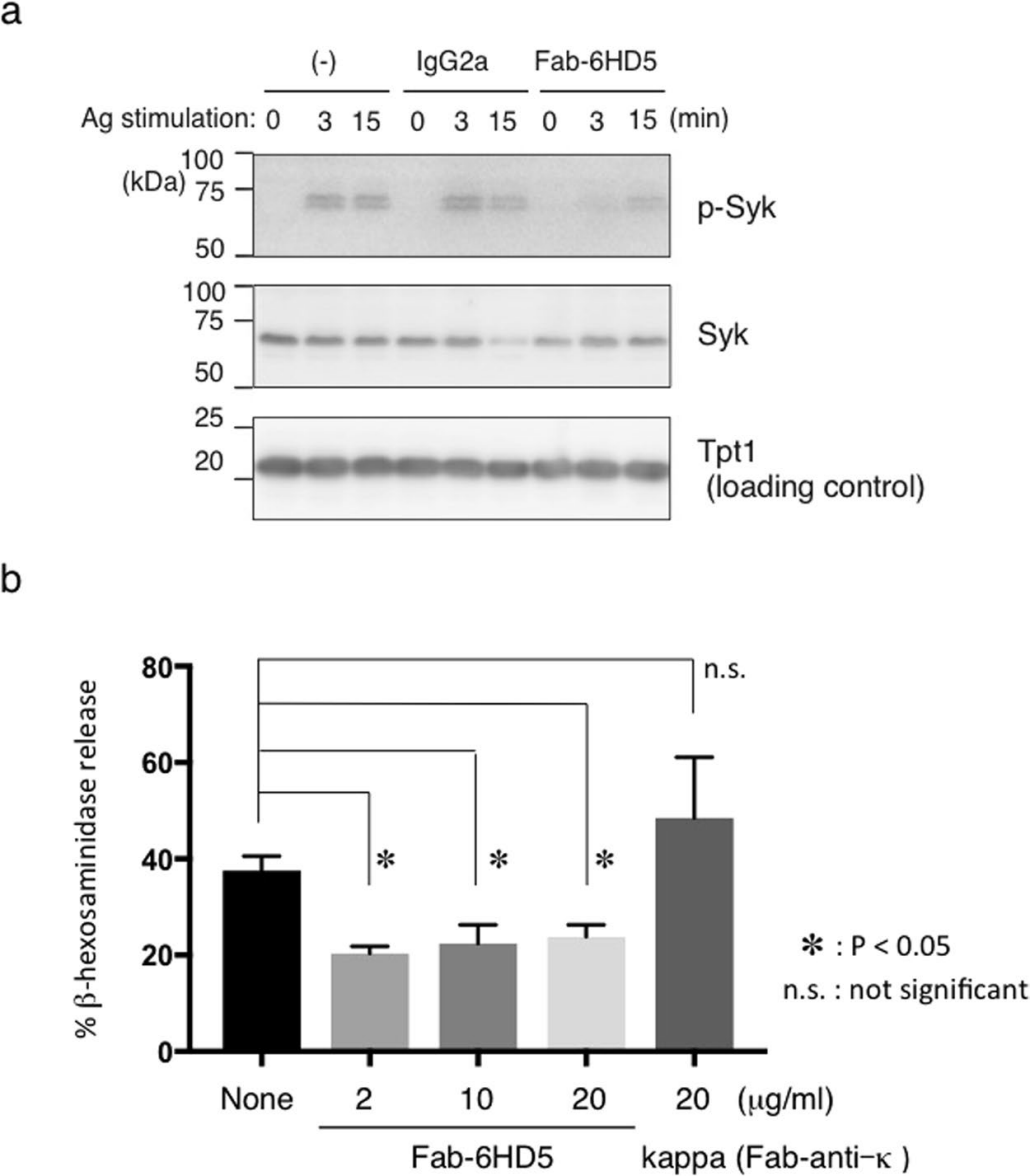
(**a**) Fab-6HD5 inhibits mast cell degranulation. RBL-2H3 cells were sensitized overnight with IgE (SPE-7) and further incubated with a serial dilution of highly purified Fab-6HD5 (2–20 μg/ml) for 2 hours at 37 °C. After washing, cells were incubated with DNP-BSA for 1 hour at 37 °C. The supernatant was then incubated with p-nitrophenyl N-acetyl-beta-D-glucosamine (PNAG) for 1 hour at 4 °C. IgE-mediated degranulation was monitored by β-hexosaminidase activity. The amount of β-hexosaminidase was determined by measuring the optical density at 405 nm. (**b**) Fab-6HD5 inhibits Syk phosphorylation. RBL-2H3 cells were incubated with anti-TNP IgE (0.5 μg/ml) for 1 hour. Cells were further incubated with highly purified 6HD5-Fab (2 μg/ml) or control IgG2a overnight. After washing, cells were stimulated with TNP26-BSA (100 ng/ml) for indicated periods and cell extracts were subjected to Western blotting. Proteins were detected with anti-phosphorylated Syk, anti-Syk, and anti-Tpt1 antibodies followed by HRP-conjugated anti-rabbit or anti-mouse antibody.

To confirm the results obtained *in vivo*, we investigated whether Fab-6HD5 could inhibit mast cell degranulation by measuring β-hexosaminidase release via spleen tyrosine kinase (Syk) activation. For this purpose, rat basophilic leukemia cells (RBL/2H3) were incubated overnight with DNP-IgE (SPE-7). Following sensitization, cells were incubated with serial dilutions of highly purified Fab-6HD5 for 2 hours at 37 °C. After washing the cells to remove excess IgE and Fab-6HD5, the cells were then stimulated with DNP-BSA to trigger degranulation. After stimulation, Syk activity and the percentage of β-hexosaminidase released were measured as described in the Methods section. Consistent with the results obtained using the *in vivo* PCA assay, our results demonstrated that Fab-6HD5 inhibits Syk activity and β-hexosaminidase release from RBL/2H3 cells in a dose-dependent manner *in vitro* (Fig. 1a,b). Notably, the optimal concentration of Fab-6HD5 (2 μg/ml) to inhibit Syk phosphorylation and β-hexosaminidase release was much lower than that of omalizumab (2 mg/ml) needed to inhibit leukotriene release in mast cells and basophils^30^. Taken together, our results obtained from *in vivo* and *in vitro* studies raise the possibility that further development of recombinant humanized anti-IgE antibodies may contribute to preventing allergic diseases with fewer side effects.

### Fab-6HD5 interacts with the IgE-FcεRIα complex on the surface of rat mast cells

**Figure 2:**
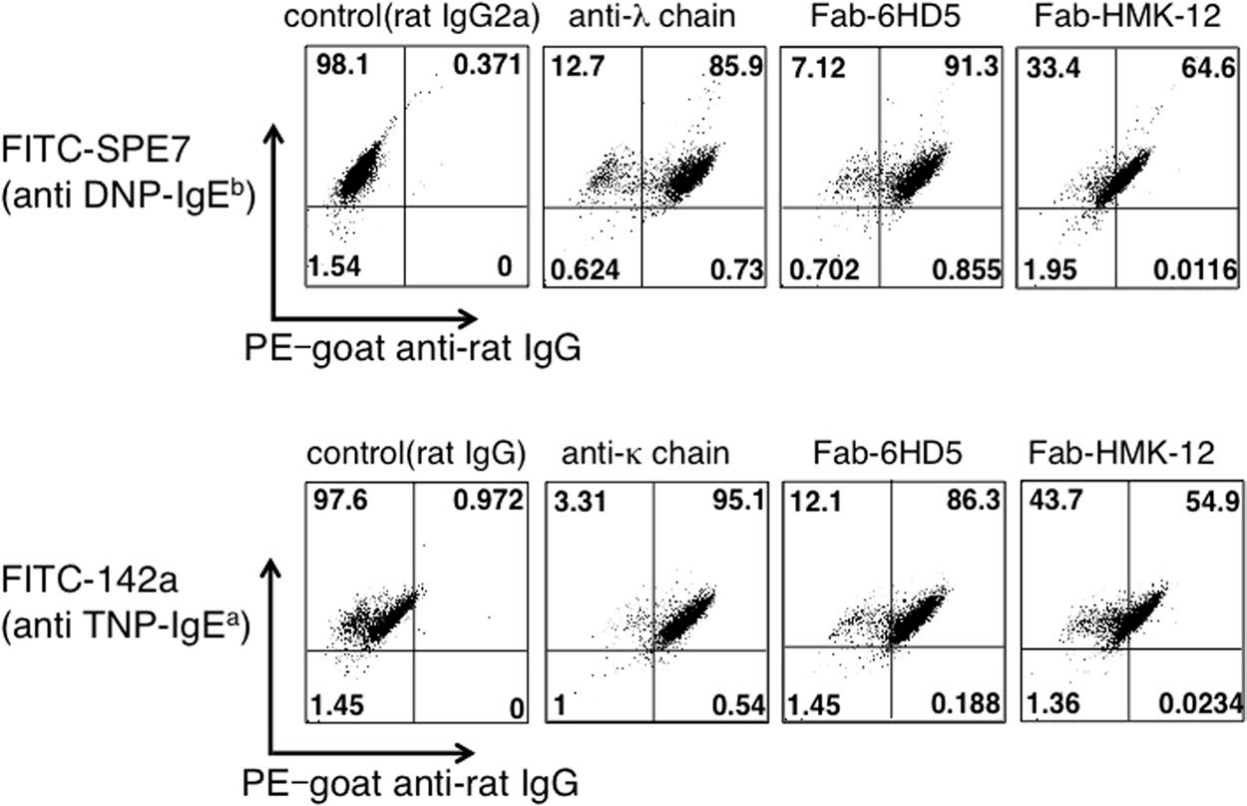
Detection of Fab-6HD5 bound to RBL-2H3 cells through flow cytometry analysis. RBL-2H3 cells were incubated with FITC-labeled SPE-7 (anti-DNP IgE) or 142a (anti-TNP IgE) for 30 min at 4 °C. After the cells were washed with PBS, they were incubated with Fab-6HD5, Fab-HMK-12 or anti-Ig light chain for 30 min at 4 °C. The cells were then incubated with PE-goat anti-rat IgG for 30 min at 4 °C and subjected to FACS analysis.

According to recent studies, several anti-IgE Fab fragments inhibit IgE-mediated serotonin release from mast cells^31,32^. These antibodies are thought to recognize the binding sites of IgE for FcεRIα. We have previously shown that the anti-IgE antibody HMK-12 also inhibits the binding of IgE to the IgE-FcεRIα complex^33^. By contrast, Fab-6HD5 appears to suppress the release of chemical mediators after IgE antigen-mediated crosslinking of surface FcεRIα, which indicates that this antibody is not a competitive inhibitor of the IgE-FcεRIα interaction. We therefore investigated the degree of binding of Fab-6HD5 and Fab-HMK-12 to the IgE-FcεRIα complex on the surface of mast cells. For this purpose, RBL-2H3 cells were first incubated with FITC-labeled SPE-7 (anti-DNP IgE). After washing, the cells were incubated with Fab-6HD5, Fab-HMK-12 or anti-Ig light chain (λ) antibodies, followed by PE-goat anti-rat IgG, and the samples were then subjected to FACS analysis. The results shown in Fig. 2 clearly indicate that SPE-7^+^ 6HD5^+^ and SPE-7^+^ λ ^+^ cells were 91.3% and 85.9%, respectively, whereas SPE-7^+^ HMK-12^+^ cells were 64.6%. Similar results were also obtained for another IgE isotype clone 142a (anti-TNP IgE), i.e., SPE-7^+^ 6HD5^+^ (86.3%), SPE-7^+^ κ ^+^ (95.1%) and SPE-7^+^ HMK-12^+^ (54.9%) cells. These results indicate that Fab-6HD5 interacts with preformed IgE-FcεRIα complex on the surface of mast cells.

**Figure 3:**
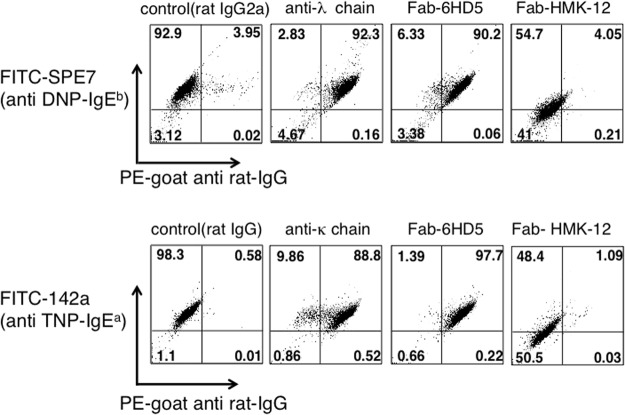
Competitive inhibition of IgE binding to FcεRI by anti-IgE antibodies. FITC-labeled SPE-7 (anti-DNP IgE) or 142a (anti-TNP IgE) was premixed with Fab-6HD5, Fab-HMK-12 or anti-Ig light chain. After incubation for 30 min at room temperature, the above mixture was added to RBL-2H3 cells and incubated for 30 min at 4 °C. After the washes, the cells were incubated with PE-goat anti-rat IgG for 30 min at 4 °C and subjected to FACS analysis.

To confirm these results, competition experiments were performed by premixing SPE-7 with Fab-6HD5, Fab-HMK-12, or anti-Ig light chain (λ) antibodies before incubation with RBL-2H3 cells (Fig. 3). Consistent with the results shown in Fig. 2, FACS analysis revealed that SPE-7^+^ 6HD5^+^ and SPE-7^+^ λ ^+^ cells were 90.2% and 92.3%, respectively, whereas SPE-7^+^ HMK-12^+^ cells were 4.05%. Likewise, similar results were obtained for 142a, i.e., SPE-7^+^ 6HD5^+^ (97.7%), SPE-7^+^ κ ^+^ (88.8%) and SPE-7^+^ HMK-12^+^ cells (1.09%). Taken together, these results suggest that Fab-6HD5 is not directed against the IgE Cε3 domain, which is involved in binding to FcεRIα^34^.

### Fab-6HD5 is directed against the IgE Cε2 domain

**Figure 4:**
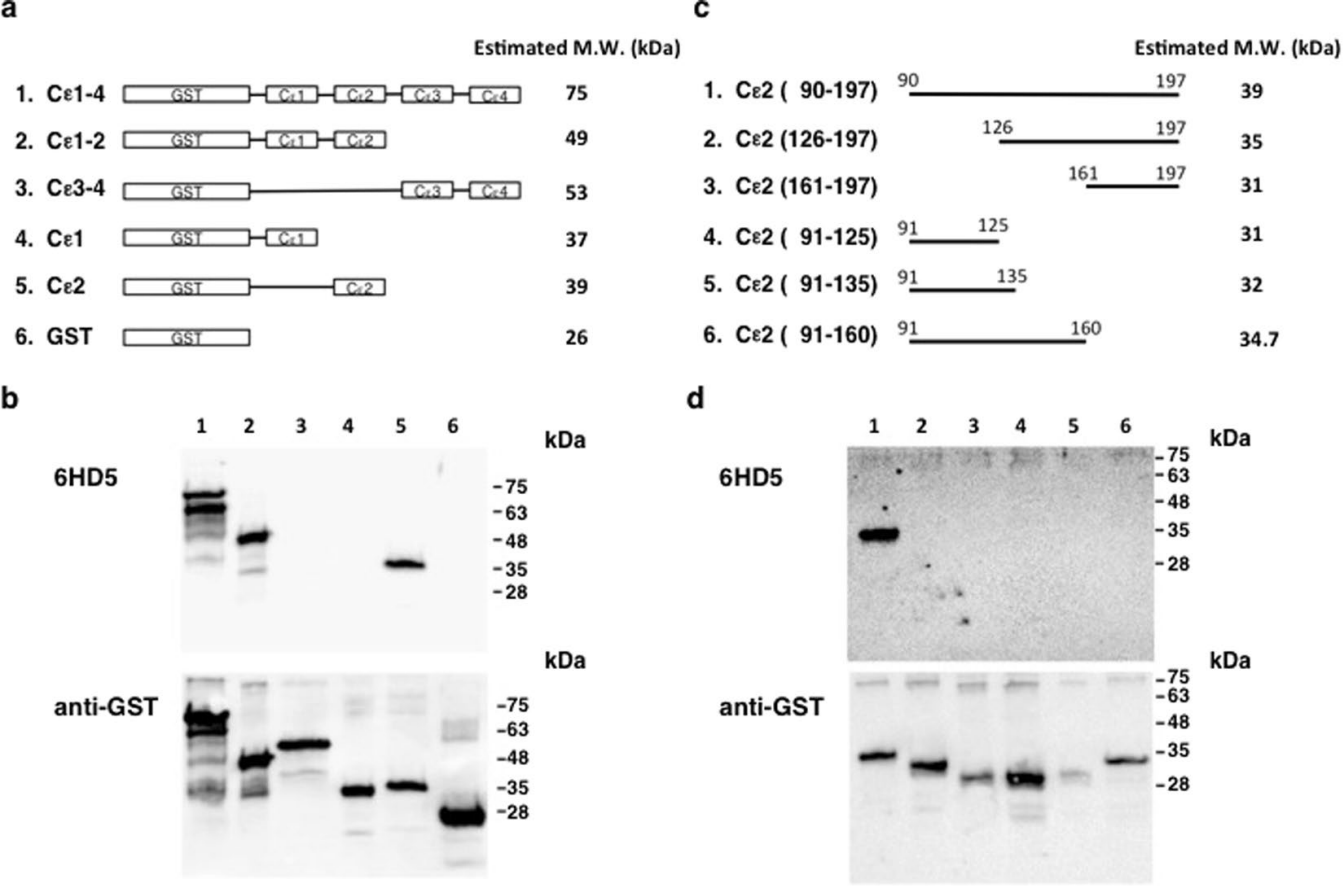
(**a**) GST-IgE H chain constant region fusion molecules. cDNA was prepared from the mouse IgE-producing hybridoma SPE-7. The resulting cDNA was amplified for the coding gene of the IgE H chain constant region (Cε1-4) using the PCR primer sets listed in Supplementary Table 1. (**b**) Epitope mapping of Fab-6HD5 against the IgE H chain constant region. GST fusion molecules containing IgE Cε domains (Cε1-4, Cε1-2, Cε3-4, Cε1, Cε2) were run on 12.5% SDS-PAGE under reducing conditions, transferred to a PVDF membrane and probed with Fab-6HD5 followed by Pox goat anti-rat IgG. Immunoreactivity was detected using an Imagequant LAS 4000. (**c)** GST fusion molecules containing IgE Cε2 fragments. cDNA was prepared from the mouse IgE producing hybridoma, SPE-7. The resulting cDNA was amplified for the coding gene of IgE Cε2 fragments using the PCR primer sets listed in Supplementary Table 1. (**d**) Epitope mapping of Fab-6HD5 against the IgE Cε2 domain. GST fusion molecules containing IgE Cε fragments were run on 12.5% SDS-PAGE under reducing conditions, transferred to a PVDF membrane and probed with Fab-6HD5 followed by Pox goat anti-rat IgG. Immunoreactivity was detected using an Imagequant LAS 4000.

Based on the above findings, we next explored identifying the IgE epitopes recognized by Fab-6HD5. IgE is structurally similar to other immunoglobulin with two heavy and two light chains. It consists of four heavy chain constant region domains (Cε1, Cε2, Cε3, Cε4) and binds to FcεRIα on mast cells and eosinophils with extraordinarily high affinity^35^. To define the epitopes recognized by Fab-6HD5, we prepared GST fusion molecules comprising IgE Cε domains (Cε1-4, Cε1-2, Cε3-4, Cε1, Cε2) (Fig. 4a). These molecules were then subjected to Western blot analysis using Fab-6HD5 (Fig. 4b). Under reducing conditions, our results clearly demonstrated that only GST fusion molecules containing the Cε2 domain (Cε1-4, Cε1-2, Cε2) reacted with Fab-6HD5, but others containing the Cε1, Cε3 and Cε 4 domains (Cε1, Cε3-4) were found to be negative. These results indicate that Fab-6HD5 is directed against the IgE Cε2 domain.

### Fab-6HD5 recognizes conformational epitopes on the IgE Cε2 domain

To further narrow down the binding epitopes on the Cε2 domain, we prepared several GST fusion molecules with Cε2 fragments (Fig. 4c). Western blot analysis revealed that Fab-6HD5 reacted with the full-length Cε2 domain (90–127), but not with a Cε2 fragment that had the N-terminal deleted (126–197) (Fig. 4d). Interestingly, neither of the Cε2 fragments with the C-terminal deleted, Cε2 (91–125) and Cε2 (91–160), reacted with Fab-6HD5, which suggests that Fab-6HD5 recognizes conformational epitopes comprised of amino acids residing on Cε2 (91–125) and Cε2 (161–197) fragments.

It had been thought that the Cε2 domain is not involved in the interaction between IgE and FcεRIα. However, deletion of the Cε2 domain from the IgE H chain constant region increased the rate of dissociation of IgE from the receptor, which suggests that the IgE Cε2 domain plays a pivotal role in stabilizing the interaction of IgE with FcεRIα^36^. These findings support our previous observations that indicate that some anti-IgE antibodies, including 6HD5, can remove an IgE molecule already bound to FcεRIα on the surface of mast cells or basophilic leukemia cells and inhibit IgE-mediated systemic or local anaphylactic reactions^33,37^.

## Conclusions

We have demonstrated that a small amount of anti-IgE antibody Fab-6HD5 strongly inhibits PCA reactions *in vivo*, as well as Syk activity and β-hexosaminidase release from rat basophilic cells *in vitro*. Flow cytometry analysis and deletion-mapping studies revealed that specific binding of Fab-6HD5 to the IgE Cε2 domain prevents allergic reactions over a long period of time through interacting with a preformed IgE-FcεRIα complex on the surface of rat mast cells. Given that the Cε2 domain plays a key role in stabilizing the interaction of IgE with FcεRIα, our findings suggest that pre-administration of a certain antibody directed against IgE CH domains may provide better options for preventing Type I hypersensitivity reactions.

## Methods

### Animals and cells

Female Sprague-Dawley rats were purchased from SLC Inc., Japan. The animals were maintained under specific pathogen-free (SPF) conditions at the animal facility of Juntendo University. To perform the experiments under the same conditions but without bias, all SPF animals were allowed to acclimate for 7 days before they were used in experiments. All animal studies were performed according to the National Institute of Health Guidelines for Care and Use of Laboratory Animals. The experimental protocol was approved by the Animal Experimentation Committee of Juntendo University (Registration Number 1181, Approval Number 260229). The rat basophilic leukemia cells (RBL-2H3) were generous gifts from Dr. C. Ra (Department of Immunology, Nippon University, Tokyo, Japan). RHL-2H3 cells were cultured in Dulbecco’s Modified Eagle Medium (DMEM) (Gibco, Carlsbad, CA) supplemented with 10% FCS and maintained in a humidified incubator (5% CO_2_) at 37 °C.

### PCA reactions

PCA reactions were performed as previously described^38,39^. Several sites of freshly shaved skin of 2 rats for each group were injected intradermally with 100 ng/0.1 ml anti-DNP-IgE (SPE-7) or anti-TNP-IgE (142a). Twenty-four hours later, 1 μg/ml of Fab-6HD5, 6HD5, Fab-HMK-12, Fab-HMK-12, rat IgG and anti-κ antibody were injected into the same sites. Two hours after the second series of injections, the rats were injected intravenously with 1 ml of mixture comprised of equal quantities of 0.5% Evans blue dye in saline and 1 mg/ml of DNP-BSA or TNP-BSA. The reactions (the diameters of the blue spots) were measured 30 min later. The experiments were repeated three times. All experiments were performed in triplicate by investigators (T.H., A.K.) who were blinded to the measurements of the PCA reactions.

### Time-course studies of PCA

Several sites of freshly shaved skin of 2 rats for each group were injected intradermally with 1 μg/ml of SPE-7 on day 0.

In the first group, serial dilutions of Fab-6HD5 were injected into the same site on day 1. Two hours later, the rats were injected intravenously with 1 ml of mixture comprised of equal quantities of 0.5% Evans blue dye in saline and DNP-BSA (1 mg/ml).

In the second group, an Evans blue extravasation assay was performed on day 10. The reactions (the diameters of the blue spots) were measured 30 min later.

### Monoclonal antibodies

SPE-7 (anti-DNP murine IgE antibody) and 141a (anti-TNP murine IgE antibody) were purchased from Sigma-Aldrich Co. LLC (USA) and Pharmingen (USA), respectively. The rat monoclonal antibodies 6HD5 and HMK-12 (specific for murine IgE) have been described previously^33,37^. The rat IgG anti-murine κ antibody and phycoerythrin (PE)-labeled goat anti-rat IgG were purchased from BioLegend (CA, USA) and Jackson ImmunoResearch (PA, USA) respectively. Fab fragments of 6HD5 and HMK12 were prepared by Immune-Biological Laboratories Co. Ltd (Gunma, JAPAN). Highly purified Fab fragments of 6HD5 were prepared using Ficin, a cysteine protease isolated from fig latex. Briefly, 6HD5 (1 mg/ml) was digested with 0.5 ml of the immobilized Ficin resin slurry (Thermo scientific prod. # 44881) twice in the presence of 25 mM cysteine. The resulting Fab-6HD5 fragments were used for *in vitro* blocking assays of Syk phosphorylation or β-hexosaminidase release.

### Antigen

The 30 molecules of DNP (70 kDa molecular weight) from bovine serum albumin were a generous gift from Dr. Z. Ovary (Department of Pathology, NYU medical center, USA). TNP-BSA and Evans blue dye were obtained from Wako Pure Chemical Industries (Tokyo, Japan).

### Flow cytometry analysis

Anti-IgE antibodies bound to RBL-2H3 cells were detected by flow cytometry analysis.

For the experiment, 5 × 10^5^ RBL-2H3 cells were incubated with 1 μg/ml of FITC-Labeled SPE-7 (anti-DNP IgE) or 141a (anti-TNP IgE) for 30 min at 4 °C. The cells were washed twice with PBS and incubated with 1 μg/ml of Fab-6HD5, Fab-HMK-12 or anti-Ig light chain antibodies for 30 min at 4 °C. After the washes, the cells were incubated with 10 ng/ml of PE-goat anti-rat IgG for 30 min at 4 °C. The cells were then washed twice with PBS and analyzed using a FACSCalibur (Becton-Dickenson, CA, USA). For competition experiments, 1 μg/ml of FITC-Labeled SPE-7 or 141a was premixed with 1 μg/ml of Fab-6HD5, Fab-HMK-12 or anti-Ig light chain antibodies. After incubation for 30 min at room temperature, the mixture was added to 5 × 10^5^ RBL-2H3 cells and incubated for 30 min at 4 °C. The cells were washed twice with PBS and incubated with 10 ng/ml of PE-goat anti-rat IgG for 30 min at 4 °C. The cells were then washed twice with PBS and analyzed using a FACSCalibur (Becton-Dickenson, CA, USA)

### β-hexosaminidase release assay

RBL-2H3 cells were seeded in 96-well flat bottom plates (2 × 10^4^ cells/well). The adherent cells were incubated with 1 μg/ml of mouse anti DNP-IgE (SPE-7) overnight at 37 °C. Following sensitization, the cells were washed and incubated with a serial dilution of highly purified Fab-6HD5 for 2 hours at 37 °C. The cells were washed three times with HEPES-buffered Tyrode’s solution, pH 7.4 (HEPES-Tyrode’s solution) to remove excess IgE and anti-IgE. Then, the cells were incubated with 100 ng/ml of DNP-BSA in 0.1% BSA-HEPES-Tyrode’s solution for 1 hour at 37 °C to trigger degranulation. After stimulation, aliquots (10 μl) of the supernatant from each well were incubated with 40 μl of substrate solution, p-nitrophenyl-N-acetyl-beta-D-glucosamine in 0.1 M citrate buffer, pH 4.5 (1.3 mg/ml) for 1 hour at 37 °C. The color formed by hydrolysis of the substrate to produce p-nitrophenolate was measured at 405 nm on a microplate reader (BioRad, Benchmark plus). The total cellular content of β-hexosaminidase was measured in 1% TritonX-100 cell lysates.

% β-hexosaminidase release was calculated as follows:

$$ \% \,{\rm{release}}=({\rm{sample}}\,{\rm{O}}.\,{\rm{D}}.-{\rm{control}}\,{\rm{O}}.\,{\rm{D}})/(100 \% \,{\rm{lysis}}\,{\rm{O}}{\rm{.D}}.-{\rm{control}}\,{\rm{O}}.\,{\rm{D}})\times 100$$

### Syk phosphorylation assay

RBL-2H3 cells were incubated with anti-TNP IgE (0.5 μg/ml) (clone C38-2, BD Biosciences) for 1 h. Highly purified 6HD5-Fab (2 μg/ml) or control IgG2a (clone eBR2a, eBioscience) was added and further incubated overnight. After washing, cells were detached with 0.05% Trypsin-EDTA, collected, washed and resuspended at 2 × 10^6^/mL in RPMI containing 0.1% BSA and 10 mM Hepes pH 7.4. Cells were stimulated with TNP26-BSA (100 ng/ml) for indicated periods and cell extracts were subjected to Western blotting. Proteins were detected with anti-phosphorylated Syk (#2710, Cell Signaling Technology), anti-Syk (#2712), and anti-Tpt1 (clone 2A3, Novus Biologicals) antibodies followed by HRP-conjugated anti-rabbit (#7074, Cell Signaling Technology) or anti-mouse (#7076) antibody.

### Preparation of GST-IgE H chain constant region fusion molecules

Total RNA was isolated from the mouse IgE-producing hybridoma SPE-7 and reverse-transcribed using oligo dT primers. The coding gene for the IgE H chain constant region (Cε1-4) and truncated regions were synthesized from the cDNA and subcloned into pGEX-6P-3 (GE Healthcare). The PCR primer sets and primer sequence information are listed as Table S1. Expression vectors for GST-IgE H chain constant region fusion molecules were introduced into the BL21(DE3) pLysS E. coli strain, and expression of the GST-fusion molecules was induced by isopropyl-β-D-1-thiogalactopyranoside (IPTG, 0.2 mM final) for 2 hours at 32–37 °C.

### Western blot analysis

Samples of GST-IgE H chain constant fusion molecules (Cε1-4) were run on 12.5% of SDS-PAGE under reducing conditions, transferred to a PVDF membrane (Millipore corp.) and probed with Fab-6HD5 followed by peroxidase-conjugated goat anti-rat IgG, F(ab’)2 fragment specific (Jackson ImmunoResearch, 1:20000). Signals were developed using Pierce ECL Plus Western Blotting Substrate (Thermo 32132). Immunoreactivity was detected with an Imagequant LAS 4000 (GE Healthcare). To remove the first antibody, the membranes were soaked in stripping buffer (2% SDS, 100 mM mercaptoethanol in 62.5 mM Tris-HCl pH 6.8) at 65 °C for 30 min with occasional shaking. After washing the membranes three times with 0.05% Tween-PBS and blocking them with BlockAce (DS Pharma Biomedical), the membranes were re-probed with anti-GST-tag pAb (MBL, 1:1000) followed by HRP-conjugated anti-rabbit IgG (Jackson ImmunoResearch, 1:20000). The ECL reaction and acquisition of chemiluminescent signals from immunoblots were performed as described above. For Syk phosphorylation assay, blots were probed with respective antibodies, anti-phosphorylated Syk, anti-Syk, and anti-Tpt1 followed by HRP-conjugated anti-rabbit or anti-mouse antibody.

### Statistical analyses

Data generated from the *β*-hexosaminidase release assay were analyzed by an unpaired *t* test using GraphPad PRISM to identify significant differences. *P* values < 0.05 were considered significant.

## Electronic supplementary material

Supplementary Information
