# Supplementary material for: The Fab fragment of anti-IgE Cε2 domain prevents allergic reactions through interacting with IgE-FcεRIα complex on rat mast cells
Source: Sci Rep. 2018 Sep 24;8:14237. doi: 10.1038/s41598-018-32200-z (PMC6155129; doi:10.1038/s41598-018-32200-z)
Supplement: Supplementary file 1 — Supplementary Information [file 41598_2018_32200_MOESM1_ESM.docx]

**The Fab fragment of anti-IgE Cε2 domain prevents allergic reactions through interacting with IgE-FcεRIα complex on rat mast cells**

Takao Hirano^1^, Akemi Koyanagi^2^, Kaoru Kotoshiba^3^, Yoichi Shinkai^3^, Masataka Kasai^4^, Tomoaki Ando^4^, Ayako Kaitani^4^, Ko Okumura^4^

& Jiro Kitaura^4^

^1^Division of Hematology, Department of Internal Medicine, Department of General Medicine, Juntendo University Nerima Hospital, 3-1-10 Nerima Takanodai

Nerimaku, Tokyo, Japan

^2^Laboratory of Cell Biology, Research Support Center, Juntendo University Graduate School of Medicine, 2-1-1 Hongo, Bunkyoku, Tokyo, Japan

^3^Cellular Memory Laboratory, RIKEN, 2-1 Hirosawa, Wako, Saitama 351-0198, Japan

^4^Atopy (Allergy) Research Center, Juntendo University Graduate School of Medicine

Juntendo University Graduate School of Medicine, 2-1-1 Hongo, Bunkyoku, Tokyo, Japan

Supplementary Information
